# Happy@Work: protocol for a web-based randomized controlled trial to improve mental well-being among an Asian working population

**DOI:** 10.1186/1471-2458-14-685

**Published:** 2014-07-04

**Authors:** Qi Yuan, Su Liu, Szehang Tang, Dexing Zhang

**Affiliations:** 1 JC School of Public Health and Primary Care, the Chinese University of Hong Kong, Shatin, Hong Kong SAR

**Keywords:** Mental health, Promotion, Positive psychology, Psychological capital, Return-on-investment, Working population, Hong Kong

## Abstract

**Background:**

Mental health issues pose a serious concern in the workplace for the huge productivity loss and financial burden associated with it. Unlike the traditional ‘fixing-what-is-wrong’ approach, positive psychology offers a less-stigmatized way to promote mental health. Psychological capital, a concept originated from positive psychology, has been proven effective in improving mental well-being and work performance. However, little evidence exists for its implementation among Asian working population or its cost-benefit for organizations adopting such promotion strategy. The current study is designed to assess the protective effects of a web-based psychology capital intervention among Hong Kong working population on individuals’ mental health and work performance, as well as organizations’ return-on-investment.

**Methods/Design:**

A two-arm randomized controlled trial design will be adopted. Eligible working adults will be randomly allocated to either the intervention group or the waiting-list control group, with 177 participants in each arm. The intervention, which consists of four web-based training sessions, each targeting one of the psychological capital components (hope, efficacy, optimism and resilience), will be implemented over a 4-week period. On-line surveys will assess the participants in each group at baseline, intervention completion, 1 and 3 months after the completion. The primary outcome is individuals’ psychological capital level; secondary outcomes include individuals’ well-being, depressive symptoms, work engagement and productivity. Return-on-investment will be calculated from the employers’ perspective based on productivity gain, savings in medical expenditure, as well as operation and time costs. Analysis will follow the intention-to-treat principle.

**Discussion:**

This is the first experimental study that explores the applicability of psychological capital development among Asian population. Through investigating changes in individuals’ work productivity from absenteeism and presenteeism, this will be one of the few studies that quantify productivity gains from any type of mental health promotion. By demonstrating effectiveness in improving mental well-being and a positive return-on-investment rate, the study may help convince more uptake of similar positive psychology interventions at workplace in Asia and elsewhere.

**Trail Registration:**

Number (assigned by Centre for Clinical Trials, Clinical Trials Registry, The Chinese University of Hong Kong): CUHK_CCT00396. Registration Date: 2014/02/13

## Background

Accounting for 5.45% of global disease burden in 2010, mental disorders have become a significant public health threat and are expected to cause even heavier burden to society in the coming years [1]. The economic loss associated with mental disorders is particularly striking among the working-age adults, as these conditions affect individuals’ work performance, short-term disability, absenteeism, as well as turnover rate [2,3]. These, in turn, affect productivity and business performance. Estimates indicate that mental disorders had cost the business sector $50.7 billion in the United States [4], $14.4 billion in Canada [5] and $14.8 billion in Australia [6] annually. Globally, the cumulative loss of economic output due to mental disorders is estimated to reach $16 trillion in the next 20 years, or 25% of world Gross Domestic Product (GDP) in 2010 [7]. Since 2000, the World Health Organization (WHO) has pointed out that there is a need among employers to recognize mental health issues as a legitimate workplace concern [8].

However, mental disorders are usually associated with stigma and discrimination. Compared with treating the diseases themselves, coping with the stigma is often reported to be more difficult [9]. In the workplace where individuals are supposed to be productive, such stigma can be worse. Employees with depression or other mental disorders may avoid assistance and effective treatment, as a result [10]. Facing this challenge, promoters of workplace mental health have been searching for an alternative more positive approach than the traditional “fixing-what-is-wrong” intervention. The rationale for positive psychology interventions (PPIs) is to nurture the strengths or positive traits of individuals, thereby buffer the negative effects of stressors and improve their mental health status. It represents a far-less-stigmatized approach, therefore, has more potential to be effective as a general strategy for prevention, particularly in the workplace. Indeed, previous studies among both general population [11] and working population [12,13] demonstrated that PPIs are effective in promoting subjective well-being, with an effect size of 0.3 [14].

Based on the principles of positive psychology and organization behavior, Luthans and colleagues further developed the concept of Psychological Capital (PsyCap) [15]. It includes four personal strengths (namely, hope, efficacy, optimism and resilience), which are theoretical based, state-like (measurable and developable) and manageable for work performance improvements [15]. The potential role of PsyCap in promoting mental health in the workplace could be better illustrated through the Conservation of Resources (COR) Theory. Under the COR theory, people seek to obtain, retain, and protect resources; and stress occurs when there is a net loss of resources, the threat of loss, or a lack of resource gain following the investment [16]. At the same time, resource gains could buffer the negative effects of resource loss and create more opportunity for further gains [17].

For employees, workplace adversities are natural circumstances for resource loss, which could then lead to stress; while PsyCap, just like human and social capital, can be considered as another resource that is developable and accumulative. Gaining such resource therefore will have a potential protective effect against future resource loss. Evidence could be found in a cross-sectional study, which reported higher PsyCap level associated with higher psychological well-being over time [18]. This is further confirmed by one meta-analysis, in which a significant positive relationship between PsyCap and desirable employee attitudes, behaviors and performance was reported [19]. However, almost all the studies that explore the relationship between PsyCap and mental health outcomes by now were observational and cross-sectional in nature. Empirical evidence based on more rigorously controlled experimental study is in much need.

### Return-on-investment from employers’ perspective

Given the large stake that employers have in avoiding the cost associated with mental disorders among staff and the potential benefit the improved productivity could bring, it is not surprising that employers, particularly large companies have started investing in programs promoting mental health at workplace [20,21]. The question is whether these programs would have positive return-on-investment (ROI). Only upon proving positive ROI and making the business case can more employers be convinced to do more on this important issue, which could in turn have bigger public health impact. There has been only one previous study that estimated a ROI of 2.7 for PsyCap development [22]. The study, however, was based on a small sample from a single company, without a direct measure of improvement in work performance. The gap left in the literature is yet to be filled.

### Mental health among working population in Hong Kong

Although cross-cultural differences in expressing, interpreting and treating symptoms of mental disorders have long been recognized, studies of psychological interventions have predominantly focused among Caucasians in America and Europe [23]. With more and more Asian countries recognizing the rising burden associated with mental disorders [24], there is an increasing need of culturally competent interventions and studies evaluating their effectiveness in this part of the world. Such need is particularly strong in Hong Kong, where an estimated 14.5- 24.6 percent of citizens have a mental disorder [25]. Specifically for working population, the *2008 Hong Kong Work and Life Balance Survey* showed that the average work week is about 49.6 hours, 25 percent more than the global standard; more than 80 percent of people said that they suffered stress, and 28% reported depression [26]. Given the increasing burden of mental disorders, the WHO suggested that priority should be put on prevention and promotion [27]. However, in Hong Kong, most of the resources remain in treatment, and only a disproportionately small amount is placed on prevention [28].

Like many Asian societies, Confucianism, with collectivism as one of its key traditions [29], is rooted in Hong Kong [30]. Although several experimental studies [31-33] demonstrated that PsyCap was developable, none was targeting Asian population. How would a PsyCap intervention work in promoting mental well-being and producing positive ROI among an Asian population that emphasizes collectivism rather than individualism, such as the working population in Hong Kong? This is the key research question we try to answer with this research protocol.

### Aims of the research

Our study aims to demonstrate the effectiveness and cost-effectiveness of a psychological capital development intervention (named “*Happy@Work”)* for improving the mental well-being among working population in Hong Kong. Through designing the intervention in an on-line platform customized for local business and social environment, we will investigate the processes by which individuals from the eastern culture may adhere to the treatment and develop PsyCap. Our research will also fill some gaps in the current literature by testing hypotheses raised yet lacking evidence, for example, can the intervention benefit depressed individuals more than non-depressed ones? In addition, we will calculate the ROI of this intervention, intended for the business sector to take further actions.

## Methods/Design

### Study design

**Figure 1:**
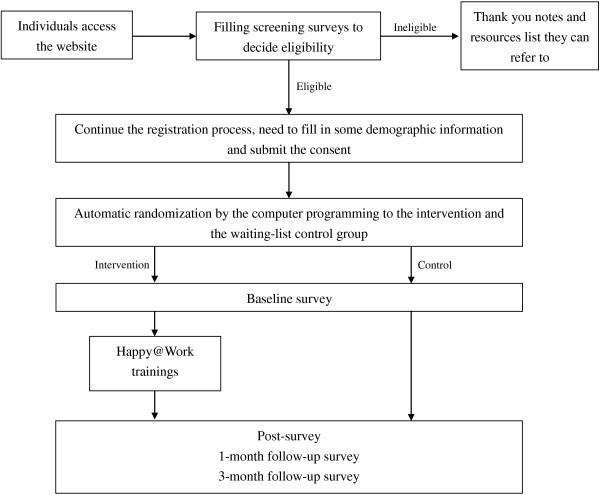
**After the screening, eligible participants will continue the registration process, followed by the baseline assessment.** The automatic randomization afterwards will then randomly allocate them into the intervention and the waiting-list control group. The intervention group participants will receive the Happy@Work trainings; while the control group will be on the waiting-list for 4 months. Both groups need to finish post-intervention, 1-month and 3-month follow-up surveys.

This study is designed as a two-arm randomized control trial. Upon visiting the intervention website, individuals will complete an initial screening to determine the eligibility. Eligible participants will continue the registration process and the baseline assessment, followed by an automatic randomization process that assigns the eligible participants into one of two groups. The intervention group will receive a 4-week access to *Happy@Work* trainings that develop each of the four PsyCap components. Measurements are scheduled at baseline, directly after the 4-week PsyCap trainings, and at 1 and 3 months follow up. The control group will be on the waiting list for 4 months before they can access the training materials. Figure 1 shows the flow of the study design.

This study is registered in the Centre for Clinical Trials, Clinical Trials Registry, the Chinese University of Hong Kong (CUHK_CCT00396). The study protocol, informed consent procedure and survey instruments have been approved by the Joint Chinese University of Hong Kong - New Territories East Cluster (CUHK-NTEC) Clinical Research Ethics Committee (CREC), under the reference number CRE-2014.001.

### Inclusion and exclusion criteria

Participants will be included if they: (1) are above 18 years old; (2) have a full-time job for at least 1 month (to ensure that we can compare work productivity before and after the intervention); (3) have access to the internet and (4) have sufficient knowledge in using computers and understanding traditional Chinese (i.e., the language the intervention program is developed in).

In order to monetize the work productivity changes, we need information such as monthly salary for participants. In this case, individuals who do not receive any monthly salary from current jobs (e.g. voluntary workers) will be excluded. Because of the ethical consideration and potential contaminations, individuals who are currently receiving professional mental health treatment or taking any psychotropic medication will be excluded as well.

### Sample size

Based on the existing evidence from PPIs, we expect to find a standard effect size of 0.3 or larger comparing the intervention group with the control group [14]. To achieve a statistical power of 80%, given a two-sided alpha of 0.05, we need at least 177 participants in each condition, i.e. a sample size of 354 or more.

### Recruitment of participants

Since the intervention is designed to cater needs among local general working population, recruitment of participants is open to the public. Advertisement about the *Happy@Work* program is put up in relevant local magazines, websites, and other public space. Anyone who meets the eligibility criteria is welcome to participate. Furthermore, to maximize the recruitment efforts and to increase awareness among employers, we will also approach them directly, targeting consent from Human Resources managers to encourage staff to participate. More specifically, we have strategically teamed up with the Employers’ Federation of Hong Kong in the beginning, so that the Federation can recruit participants from its member organizations (mostly companies of large sizes). We will also contact small-medium enterprises associations and conduct workshops for specific companies through professional networks to further promote the program. During recruitment, we will highlight the potential benefits of the program, appealing to different audiences. For employers, we emphasize its effect on increasing morale and productivity, while reducing turn-over and medical cost; for individuals, we highlight the benefit of improving well-being and career development, while managing stress.

High dropout rate is a challenge often faced by web-based studies. We use several strategies and incentives to mitigate this problem: 1) Email reminders will be sent out to remind participants at different stages (e.g. upon availability of each training session) to adhere to the process and complete all trainings and assessments on schedule; 2) Conditioning on finishing all the assessments, an electronic certificate for participation will be awarded to participants who have successfully completed all four training sessions; 3) a HK$50 cash coupon will be offered to the first 500 participants (in either control or intervention group) who complete all the assessments.

### Description of intervention

#### Intervention content

*Happy@Work* is an individualized self-learning web-based program. The content of the intervention is designed based on a thorough literature review on PysCap [22,32-41]. It includes four training sessions, each targeting one of the four PsyCap components (Table 1): 1) hope, where a goal-setting training will be provided, mainly teaching individuals how to set up SMART (Specific, Measureable, Achievable, Relevant and Time-bound) goals [31-33]; 2) efficacy, an expressive writing training, which requires individuals to write about their personal feelings on their own work, past mastery experiences on work-related issues, etc. [35,42]; 3) optimism, which teaches individuals the ABCDE model (Adversity, Belief, Consequences, Disputation and Energization) of “learned optimism” [41]; and 4) resilience, where the risk management and resource leverage practice skills will be trained [31-33].

**Table 1:** Content of the intervention program

**Components**	**Proposed training**
Hope	Goal setting (i.e. 1. Choosing of a personal goal; 2. Creating sub-goals; 3. Pathways generation; 4. Obstacle planning)
Efficacy	Expressive writing (i.e. adapted from existing guideline, basically requires individuals to write about their personal feelings on their own work; past mastery experiences on work-related issues; vicarious learning experiences; as well as the verbal persuasion experiences)
Optimism	Learned optimism (i.e. ABCDE model: 1) A stands for adversity; 2) B for the beliefs one automatically has when it occurs; 3) C for the usual consequences of the belief; 4) D for the disputation of individuals’ routine belief; 5) E for the energization that occurs when individuals dispute the belief)
Resilience	Risk management & Resource leverage practice (i.e. evaluating the risk for an adversity; practicing using different perspective to view it; planning strategies to control it; figuring out resources available and methods of getting more helpful resources; utilizing the resources to deal with the adversity)

In order to adapt the intervention to the local business and culture environment, we held a small focus group talking to four human resources managers of local companies (3 from large firms in banking, communication, and professional service, respectively and 1 from small-medium enterprises). We asked the managers to describe the key issues staff faces in the day-to-day operations, the common sources of stress or challenges, and what a “star” employee would be like. Based on the discussion, we developed cases for typical types of adversity local workers are expected to experience, thus demonstrate how each of the PsyCap component can be utilized.

#### Intervention delivery

A web-based format will be adopted to deliver the whole intervention and conduct assessment. Compared with traditional approaches, such as face-to-face counseling, a web-based approach has several advantages: 1) relatively cheaper; 2) once developed, it may be sustained and maintained with little cost to serve more people; 3) more flexible for individuals, in the sense that they can choose wherever or whenever to access the program; and 4) the web-based data collection would obviate data entry by the researcher (and associated human error). In terms of efficacy, one meta-analysis found that web-based instruction has similar effects as classroom instruction on teaching both declarative knowledge and procedural knowledge [43]. As for data quality, previous studies have shown that data collected via internet are just as diverse as data collected through traditional methods, and participants in web-based studies would treat the study the same or to provide accurate information as participants in traditional samples [44].

We worked with a web design vendor to combine the skill trainings and the localized cases through narrative technology such as flash animation. Each session is delivered in a pseudo-classroom setting, with the same trainer (an animated character) mainly providing the knowledge and guiding participants through the trainings. In addition to the participant, another animated character (different in each session) will act as the classmate who will present the specific case developed above as his or her own work challenge, and together with the participant, try to apply the skill learned. By providing such a role model for each session, we aimed to improve the learning efficiency of participants. Once participants start a session, the flash animation will automatically play by itself, with stops built in for either a “whiteboard” function or “check-point” questions. The “whiteboard” function is designed to display key knowledge/training points in a more self-controlled pace (with move-back or move-forward buttons); while the “check-point” questions (multiple-choice or brief answer questions) are built in as “breaks” during the class, which also allow participants to be tested on knowledge gained. Generally, it takes 15–30 minutes to complete each session. To better motivate participants, “happy coins” (virtual money value) are rewarded when they correctly answer the “check-point” questions or finish “homework”, the latter consists of post-session exercises participants can access after completing the training to reinforce skills learned.

Four training sessions are released one by one on a weekly base in a preset order (i.e. hope - efficacy - optimism - resilience), so that following each session individuals could have a week to practice the learned skills. A timer linked to each participant’s password-protected account is used to control individual progress. Upon completion of baseline assessment, the timer will start. Participants have up to a week to complete each training and practice the skills learned through accessing “homework”, and they can always revisit the previous sessions. Automatic email reminders will be sent out two days after a training session is available but not yet accessed. This is scheduled to help participants better follow through the whole intervention, which will take four weeks after baseline assessment.

#### Control group

A waiting-list control condition will be employed. After the baseline assessment, the control group will receive a note informing them that they need to wait for four months before accessing the training materials. During the four-month waiting period, we will send email reminders to remind them to finish the post/follow-up surveys. As an incentive, the control group is also eligible to receive cash coupons upon completing all assessments.

### Outcome assessment

The primary outcome is the level of psychological capital. The secondary outcomes include well-being, depressive symptoms, work engagement and economic cost-benefit (as measured by work productivity, medical expenditure and time cost for using the website). All these measures are self-reported, and will be collected through the *Happy@Work* website. Assessments will be made at the baseline, immediately after individuals finish the 4-week trainings, and at 1 and 3 months follow-up after the completion. All the measurements, except specified, will be assessed in both control and intervention groups.

### Mental health status measures

The level of psychological capital will be assessed by the 24-item psychological capital questionnaire (PsyCap-24) [15]. It includes 6 items for each of its 4 components (hope, efficacy, optimism and resilience). Participants will be asked to rate the items using a 6-point scale, ranging from 1 (strongly disagree) to 6 (strongly agree). A higher score indicates higher PsyCap level. The PsyCap-24 has already been translated into Chinese and adapted in a previous Hong Kong study, showing excellent internal reliability (Cronbach’s alpha equals to 0.92) [45].

Well-being will be measured by the Warrick-Edinburgh Mental Well-being Scale (WEMWS) [46] and the Satisfaction with Life Scale (SWLS) [47], both are already available in Chinese. The WEMWS is a 14-item self-rating scale with item scores ranging from 1 to 5. The SWLS only has 5 items, where participants are asked to rate on a 7-point Likert scale, ranging from 1 (strongly disagree) to 7 (strongly agree).

Depressive symptoms will be measured by Centre for Epidemiologic Studies Depression Scale (CES-D) [48,49]. The CES-D includes 20 items, using a 4-point Likert scale (ranging from 0 to 3) to rate individuals’ depressive symptoms. It has been validated in Hong Kong before [50]. Score ranges from 0–60, with higher scores indicating more severe depression. A score of 16 to 26 for CES-D is considered as potential mild depression, and 27 or above as major depression.

### Work engagement and ROI related measures

The Utrecht Work Engagement Scale - shorten version (UWES) [51] will be used to measure engagement. It is a 9-item self-rating scale with item scores from 0 to 6. It has an excellent internal consistency (e.g. Cronbach’s alpha varied between 0.85 to 0.92 across different samples from 10 countries [52]).

Work productivity changes will be measured by the WHO’s Health and Performance at Work Questionnaire - short form (HPQ) [53], which obtains information on individuals’ past 4 weeks productivity through assessing both absenteeism and presenteeism. It is currently unavailable in traditional Chinese. A standard ’translation and back-translation’ process was adopted to translate it into traditional Chinese [54]. We pre-tested the Chinese version before making further improvement.

The other two components for calculating the ROI besides work productivity are medical costs and time costs. To obtain these two measures, participants in both control and intervention groups will be asked to report the past-month medical expenditures and the share paid by their employers (if any); while the intervention group alone will also report time they spend on the entire trainings.

### Demographics and other variables

The demographic information will be collected during the registration. In the post-intervention survey, participants of the intervention group will be asked to report their satisfaction level towards our program; a manipulation check question will also be employed to explore the potential moderation effect between individuals’ self-rated devotion level and other individual-level outcomes. The control group participants, on the other hand, will be asked to report whether they have been exposed to any educational information related to mental health or psychology during the past four weeks.

### Treatment adherence and user experience

Because this is the first on-line positive psychology intervention conducted in Hong Kong and among the few in Asia, we will also evaluate the process to better understand user experience and to test the extent to which treatment is adhered and intervention is successfully implemented. In addition to the self-rated devotion level, we will collect timing and progress information (built in behind the scene as part of the system record), while participants log in every time and move along each training session, answer “check-point questions” and complete “homework”. The amount of “happy coins” itself could serve as another indicator of participants’ level of commitment to the learning experience. As part of the post-intervention assessment, we ask participants to leave open-ended comments. For those who quit the program, we plan to conduct some follow-up interviews to better understand their experience and the reasons behind the drop-out.

### Data analysis

Results will be reported according to the Consolidated Standards of Reporting Trials (CONSORT) statement regarding eHealth [55]. The primary data analysis will follow the intention-to-treat principle and compare the differences in the above outcome measures between the intervention group and the wait-list control group. Because of the potential dropout, missing data is expected. When appropriate, we will use the expectation-maximization (EM) method to impute missing data [56]. In addition, per-protocol analysis will also be conducted.

The two groups will first be described in terms of their baseline characteristics. The primary data analysis will compare the effectiveness of *Happy@Work* in the intervention group with the changes in the control group, based on the differences on PsyCap-24, WEMWS, SWLS, CES-D and UWES. Repeated measures of ANOVA will be used to check the multivariate main effects of intervention (compared with control) and time (pre, post, follow-up), as well as their interaction effect. A two-sided P value of 0.05 or less will be considered as significant. We will compute the standardized effect size (Cohen’s d) as well. Moderator analysis will also be conducted to explore which sub-group/s would benefit more from the intervention, by regressing outcome variables on independent variables like age, gender, education and baseline CES-D scores. Work productivity change will be compared between these two groups as well. SPSS V.18 will be used for the data analysis.

To make the business case for similar type of interventions, return-on-investment of the program will be calculated from the employers’ perspective using the following formula:

ROI = (Gain from investment - Cost of investment)/Cost of the investment;

where, *Gain from investment* = total monetized value of employees’ work productivity gains + savings in employers’ share of medical cost; and

*Cost of investment* = Program designing and operation costs (assuming employers self-fund similar programs) + the monetized time cost (if employees use work time for the program training*).*

Self-reported monthly salary will be used to monetize the work productivity changes and time cost for each individual. All the afore-mentioned items will be the incremental value compared with the control group.

## Discussion

### Strengths and limitations

To the best of our knowledge, this is the first experimental study that explores the applicability of PsyCap development among Asian population; and it is also one of the few studies that measure improvement in depression and mental well-being as a result of PsyCap development. We will also examine differences in these outcomes between individuals with different level of depressive symptoms. Through the investigation of changes in individuals’ work productivity from absenteeism and presenteeism, our study will be one of the few studies that quantify productivity gains and return-on-investment from any type of mental health promotion.

There are also several limitations that we need to mention. Compared with group based or individual based consultation, although web-based self-administrated interventions are usually cheaper, it might also be less effective. Meanwhile, since we will mainly use self-report method to collect data, this might cause recall bias. The ROI estimation will be less objective compared with those studies that choose one specific company and use the company records (e.g., medical claims, sick leave days) for such calculation. Another limitation would be the possible high dropout rate for this study, despite self-selection and other strategies to reduce it (e.g. email reminders and other incentives). Although studies have found that self-selected people enjoyed more obvious effects than non-self-selected individuals in PPIs [57], this also calls for caution against over-generalizing our results.

### Future implementation

If through this study, we are able to prove the intervention not only effective in improving the mental well-being of working individuals, but also bringing positive return-on-investment for employers, further efforts to improve the program, or customize it for specific industry or company is anticipated. Our research partner, the Employers’ Federation of Hong Kong would assist in this process and further funding may be sought. What’s more, a network of public and private organizations interested in the workplace mental health issues is being developed, as a side product of our recruiting efforts. We will disseminate research findings to this network and more broadly to the public. As a result, more organizations (especially those with large number of employees working under stressful condition) may be willing to invest more in similar initiatives or customize *Happy@Work* to fit their own needs in the future.

## Abbreviations

GDP: Gross domestic product; WHO: World health organization; PPIs: Positive psychology interventions; PysCap: Psychological capital; COR: Conservation of resource theory; ROI: Return-on-investment; PsyCap-24: The 24-item psychological capital questionnaire; WEMWS: Warrick-edinburgh mental well-being scale; SWLS: Satisfaction with life scale; CES-D: Centre for epidemiologic studies depression scale; UWES: The Utrecht work engagement scale - shorten version; HPQ: The WHO’s Health and Performance at Work Questionnaire; CONSORT: Consolidated standards of reporting trials; EM: Expectation-maximization.

## Competing interests

The authors declare that they have no competing interests.

## Authors’ contributions

QY and SL conceived the study, and participated in its design. QY drafted the manuscript. SL is the principle investigator for this study and she revised the manuscript. SHT contributed to the design of the intervention program. DXZ contributed to the research protocol and the data analysis plan. All authors read and approved the final version of the manuscript.

## Pre-publication history

The pre-publication history for this paper can be accessed here:

http://www.biomedcentral.com/1471-2458/14/685/prepub
